# Bovine Intestinal Alkaline Phosphatase Reduces Inflammation After Induction of Acute Myocardial Infarction in Mice

**DOI:** 10.4021/cr81w

**Published:** 2011-09-20

**Authors:** Danielle Fiechter, Suzanne Kats, Ruud Brands, Ben van Middelaar, Gerard Pasterkamp, Dominique de Kleijn, Willem Seinen

**Affiliations:** a Institute for Risk Assessment, University Utrecht, Utrecht, The Netherlands; b Department of Cardio-Thoracic Surgery, Maastricht University Medical Centre, Maastricht, The Netherlands; c Department of Experimental Cardiology, University Medical Centre Utrecht, Utrecht, The Netherlands

**Keywords:** Acute myocardial infarction, Alkaline phosphatase, Lipopolysaccharide, Inflammatory response

## Abstract

**Background:**

There has been increasing evidence suggesting that lipopolysaccharide or endotoxin may be an important activator of the innate immune system after acute myocardial infarction. Bovine intestinal alkaline phosphatase reduces inflammation in several endotoxin mediated diseases by dephosphorylation of the lipid A moiety of lipopolysaccharide. The aim of this study was to investigate the effect of bovine intestinal alkaline phosphatase on reducing inflammation after acute myocardial infarction.

**Methods:**

Just before permanent ligation of the left anterior descending coronary (LAD) artery to induce acute myocardial infarction in Balb/c mice, bovine intestinal alkaline phosphatase (bIAP) was administrated intravenously. After 4 hours, mice were sacrificed and the inflammatory response was assessed. Acute myocardial infarction induced the production of different cytokines, which were measured in blood.

**Results:**

Treatment with bovine intestinal alkaline phosphatase resulted in a significant reduction of the pro-inflammatory cytokines IL-6, IL-1β and the chymase mouse mast cell protease-1. No difference in the production of the anti-inflammatory cytokine IL-10 was observed between the control group and the bovine intestinal alkaline phosphatase treated group.

**Conclusion:**

In a mouse model of permanent LAD coronary artery ligation, bIAP diminishes the pro-inflammatory responses but does not have an effect on the anti-inflammatory response in the acute phase after acute myocardial infarction.

## Introduction

Lipopolysaccharide (LPS), an endotoxin present in the outer cell wall of Gram-negative bacteria, is a potent stimulator of the innate immune response. When entering the circulation, LPS binds to the lipopolysaccharide-binding protein (LBP) which interacts with CD14, MD2, and Toll-like receptor 4 (TLR4) to start a signaling cascade leading to cytokine production [1-4]. Cardiogenic shock is the leading cause of death among patients hospitalized with acute myocardial infarction (AMI). It is well known that AMI is associated with an increased systemic and local inflammatory response [5]. There is growing evidence suggesting that endotoxin is an important stimulus for this phenomenon. Decreased cardiac function reduces bowel perfusion, leading to hypoperfusion and ischemia of the intestinal mucosa. This results in increase of gut permeability, and subsequent translocation of endotoxin into the circulation [6, 7]. Several studies with patients in heart failure as a result of cardiogenic shock, irrespective of etiology, have shown an increase of soluble CD14 (sCD14) in plasma, TLR4 expression on monocytes and increased levels of bacteria or endotoxin when compared to control groups [6, 8-12]. Furthermore, elevated procalcitonin levels correlated to IL-6 levels have been described after acute myocardial infarction. Bacterial toxins are by far the most potent trigger for elevated procalcitonin levels [13]. Taken together, these data lead to suggest that endotoxin release is an important mediator in the observed inflammatory response after AMI.

There is increasing evidence that alkaline phosphatase is able to remove one phosphate group from the lipid A moiety of LPS, thereby dephosphorylating and detoxifying LPS [14, 15]. In mice, infected with a lethal dose of Gram-negative bacteria, mortality was reduced after injection of human placental alkaline phosphatase (HPLAP) or bovine intestinal alkaline phosphatase (bIAP) [16, 17]. A reduction in the inflammatory response induced by LPS could be observed in mice and piglets after treatment with HPLAP or bIAP [16, 18]. Oral treatment of rats with LPS resulted in a prolonged endotoxemia after inhibition of endogenous intestinal alkaline phosphatase [19]. In addition, the potential effects of alkaline phosphatase on LPS-mediated diseases have been demonstrated in animal studies with polymicrobial sepsis. Cytokine response and neutrophil influx in secondary peritonitis in mice was attenuated by bIAP [20]. Hepatic and pulmonary injury after liver ischemia-reperfusion with partial resection was reduced in rats treated with bIAP when compared to control animals [21]. Studies performed by the group of Vincent et al with bIAP administration to sheep, injected with an ultimately lethal dose of feces to mimic severe endotoxemia conditions, showed a decrease in IL-6 concentrations and a prolonged survival time [22]. In the study reported here, the left anterior descending (LAD) coronary artery ligation was used as a model to induce an AMI in mice. The primary endpoint was to examine the potential effect of bIAP on reducing the pro-inflammatory response principally depicted by IL-6 release in the acute phase after AMI by its ability to detoxify LPS. At the time point of peak IL-6 release complementary measurements of pro-inflammatory cytokines TNFα and IL-1β, and anti-inflammatory cytokine IL-10 were performed. Prior to LAD ligation, bIAP was used as a prophylaxis by intravenous administration. The resulting systemic inflammatory response was investigated.

## Methods

### Induction of acute myocardial infarction

Animals used in the present study were treated in conformity with the Guide for the Care and Use of Laboratory Animals published by the National Institutes of Health (NIH Publication No. 85-23, 1996). The study was approved by the animal ethics committee of the Faculty of Veterinary Medicine, Utrecht University. Specific pathogen free (SPF) female BALB/c mice (23-27 gram) were purchased from Charles River (Sulzfeld, Germany) and were acclimatized for 1 week under barrier conditions in filter-topped macrolon cages with drinking water and standard food pellets ad libitum. Mice were anaesthetized by inhalation of a mixture of O_2_ air and 4% isoflurane, endotracheally intubated, and mechanically ventilated. The LAD coronary artery was exposed via a left thoractomy and double ligated with an 8.0 prolene suture, as described by Salto-Tellez et al [23]. To determine at which time point after AMI induction pro-inflammatory cytokine production could be detected, mice were sacrificed at 4, 6, and 24 hours respectively after AMI (n = 3 mice per time point) induction and blood was collected.

### Bovine intestinal alkaline phosphatase

Clinical grade bovine intestinal alkaline phosphatase was obtained from Biozyme (Blaenavon, UK). One unit is defined as that amount of bIAP able to hydrolyse 1 µmole of p-nitrophenyl phosphate per minute using a Tris-glycin buffer at pH 9.6 at 25 °C.

To examine the effect of bIAP, mice were divided into two groups: an AMI group treated with bIAP (n = 4) and an AMI control group (n = 4). BIAP was injected into the tail vein just before induction of anesthesia as a single intravenous dose of 5 IU in 100 µL phosphate buffered saline (PBS) (approximately 30-50 times above plasma levels). Control mice were injected with an equal volume of PBS. Mice were sacrificed and blood was collected. Heart, lung, liver and kidneys were removed and fixed in 4% para-formaldehyde in PBS.

### Determination of alkaline phosphatase activity

Five µL of serum was incubated for 60 minutes at 37 °C with 200 µL assay mix containing incubation buffer (0.025 M glycin/NaOH, pH 9.6), p-nitrophenyl phosphate and MgCl_2_ at final concentrations of 1.25 and 2 mM respectively. The end product p-nitrophenol was quantatively determined by measuring the extinction at 405 nm.

Blood samples were centrifuged and serum was collected for determination of mouse IL-6. At the time point of peak IL-6 release complementary measurements of TNF-α, IL-1β and IL-10 protein were performed. by commercially available ELISA kits according to the manufacturers’ protocol (IL-6 and TNF-α from Biosource, Etten-Leur, The Netherlands; IL-1β from R&D Systems, Abingdon, UK; and IL-10 from BD Biosciences, Erembodegem, Belgium). After AMI in mammals, mast cells are activated to release chymases. Activation of mast cells in mice can be measured by the release of the mouse mast cell protease-1 (mMCP-1) chymase. MMCP-1 ELISA was from Moredun (Midlothian, Scotland, UK) and performed according to the manufacturer’s instructions.

### Statistics

All data presented are mean ± SEM. Statistical analysis was performed using Student’s t-test for unpaired data (GraphPad Prism). Values were considered significant when P < 0.05.

## Results

### Determination of the IL-6 response

**Figure 1:**
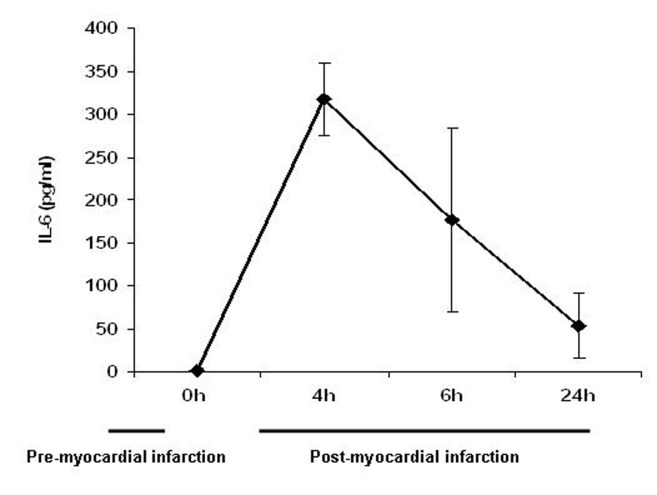
Production of the pro-inflammatory cytokine IL-6 after acute myocardial infarction. Mice were sacrificed at different time points and IL-6 production was determined (n = 3 per time point). Values are depicted as mean ± SEM.

In 9 mice it was determined at which time point after AMI in Balb/c mice IL-6 production could be detected. Before operation, IL-6 concentration was below detection limit (< 4 pg/mL) (Fig. 1). Peak IL-6 serum levels were observed 4 hours after AMI. Elevated serum levels of IL-6 could still be detected 6 and 24 hours after AMI. Based on these results, mice were sacrificed 4 hours after AMI in the bIAP treatment experiments.

### Bovine alkaline phosphatase activity

**Figure 2:**
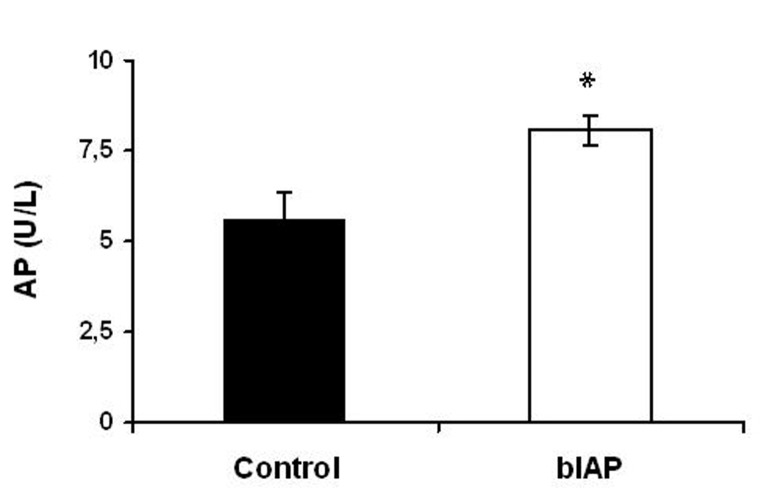
Alkaline phosphatase activity 4 hours after acute myocardial infarction. Values are depicted as mean ± SEM (n = 4 per treatment group). * P < 0.05 versus control

Alkaline phosphatase activity was determined in serum samples by measuring hydrolysis of p-nitrophenyl phosphate by alkaline phosphatase. All mice that received bIAP had slightly elevated serum levels of alkaline phosphatase activity 4 hours after AMI compared to control mice (P < 0.05). The amount of alkaline phosphatase administered was about 30-50 times total plasma level. As reported earlier by Beumer et al [16]. The total amount of administered bIAP falls within 10 minutes, thus the plasma level at timepoint of 4 hours represents a confirmation on successful intravenous administration of bIAP via the tail vein (Fig. 2).

### Cytokine response

**Figure 3:**
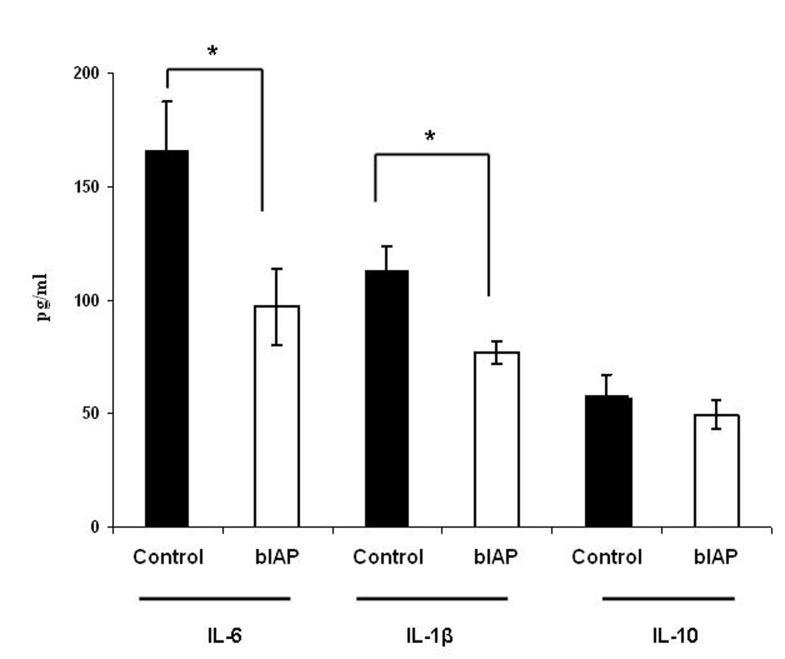
Effect of bIAP on the production of the pro-inflammatory cytokines IL-6 and IL-1β and on the anti-inflammatory cytokine IL-10 4 hours after acute myocardial infarction. Levels of IL-6, IL-1β, and IL-10 were determined using specific ELISA; (■) control mice and (□) bIAP-treated mice. Values are depicted as mean ± SEM (n = 4 per treatment group). * P < 0.05 versus control.

Before LAD coronary artery ligation, concentrations of the different cytokines were below detection levels. In contrast, 4 hours after AMI IL-6, IL-1β and IL-10 were excessively produced (Fig. 3). TNF-α production could not be determined at this time-point. Administration of bIAP resulted in a significant reduction of the pro-inflammatory cytokines IL-6 and IL-1β as compared to controls. IL-6 levels were reduced by approximately 40% and IL-1β levels by approximately 30%. No difference in the anti-inflammatory cytokine IL-10 production could be observed between the control group and the bIAP-treated group.

### Mast cell activation

**Figure 4:**
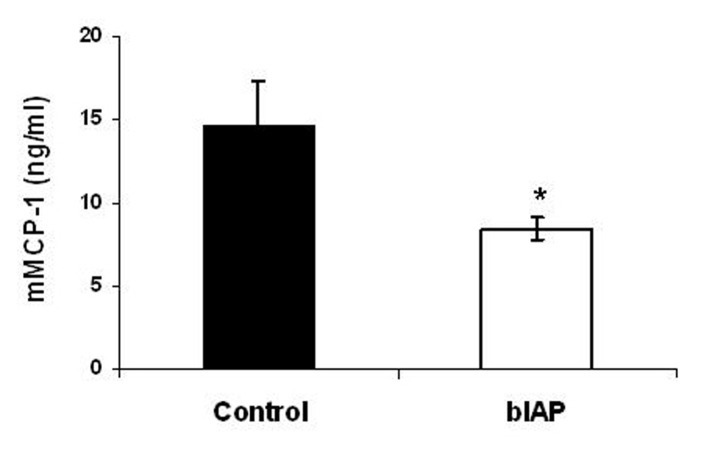
Effect of bIAP on the production of mMCP-1, 4 hours after acute myocardial infarction. Values are depicted as mean ± SEM (n = 4 per treatment group). * P < 0.05 versus control.

Serum levels of mMCP-1 were 14.7 ng/mL 4 hours after LAD coronary artery ligation. BIAP treatment reduced mMCP-1 levels in serum to 8.4 ng/mL (approximately 40%), implying a significant reduction in mast cell activation (P < 0.05) (Fig. 4).

## Discussion

Cardiogenic shock is the major cause of death in patients hospitalized with acute myocardial infarction (AMI) [24]. AMI results in intestinal hypoperfusion, which leads to increased gut permeability. Consequently, translocation of endotoxin into the circulation occurs. There has been growing evidence that presence of endotoxin is responsible for the observed systemic inflammatory response after AMI and that this may play an important role in the onset of cardiogenic shock [6, 7, 12, 13]. Reducing inflammation after AMI has received little attention in research, and a specific pharmacologic treatment to reduce the inflammatory response after AMI has yet to be introduced.

To date, several studies have demonstrated the potential therapeutic effect of bIAP on LPS-mediated diseases, and it was therefore interesting to examine the ability of bIAP to reduce inflammation after AMI [20-22]. Therefore, Balb/c mice received an intravenous injection of bIAP just before AMI induction by permanent ligation of the LAD coronary artery.

Four hours after AMI, a significant reduction in the concentrations of the two most prominent pro-inflammatory cytokines present in serum in the acute phase after AMI, IL-6 and IL-1β, was observed when compared to non-bIAP treated controls. TNF-α concentration in serum, generally believed to be an early-onset pro-inflammatory cytokine, was below detection limit, suggesting that the chosen time point is not relevant to detect this cytokine in Balb/c mice after LAD coronary artery ligation. A reduction in pro-inflammatory cytokine production indicates a diminished systemic innate immune response, which may decrease myocardial dysfunction and reduce the development of cardiogenic shock after AMI [8]. It is well known that inhibition of the complement-dependent inflammatory response, responsible for cellular alterations associated with irreversible myocardial injury, limits the extent of myocardial infarcts [25, 26]. Thus, we performed a CH50 cell lysis assay to exclude the effect of bIAP on the alternative complement pathway. That assay demonstrated that high doses of bIAP (181 U/mL) resulted in an inhibition of complement of 34%. In the low dose bIAP we used in the current study no inhibition of complement could be measured (data not shown). BIAP treatment had no effect on IL-10 production. Since IL-10 is a potent anti-inflammatory cytokine, and several in vivo studies have shown its protective role in a variety of pathological states (e.g. colitis, hepatic ischemia/reperfusion and myocardial ischemia/reperfusion), a reduced production due to bIAP treatment would not be favorable [27-29]. Chymases are abundantly produced after AMI in mammals, and are known to be involved in the cleaving of angiotensin I to form angiotensin II [30, 31]. The excessive formation of angiotensin II, which is observed in the acute phase after AMI, is arrhythmogenic, and several studies in different animal models have shown that decreasing angiotensin II formation by a specific chymase inhibitor contributes to a reduction in mortality rate in the acute phase after AMI [32, 33]. Studies in rats revealed that production of the rat chymase MCP-2 (rMCP-2) is increased after stimulation of mast cells with LPS [34, 35]. Given that bIAP has an effect on decreasing LPS toxicity, the influence of bIAP on the formation of the mouse chymase mMCP-1 was determined. In bIAP-treated mice, mMCP-1 production was significantly reduced by approximately 40% when compared to non-bIAP treated mice. This might result in a reduction of angiotensin II formation and consequently a decrease in arrhythmias, which may improve cardiac function and reduce cardiogenic shock complications [36].

Direct effects of bIAP on LPS detoxification could not be determined in this study. Since it is reported that bIAP is able to detoxify LPS through dephosphorylation of the lipid A moiety, the Limulus amoebocyte lysate (LAL) assay cannot be used as it is unable to make a discrimination between lipid A and monophosphoryl lipid A (MPLA) [37]. Therefore, decreased activation of the innate immune response because of bIAP administration could not be linked to decreased LPS levels in this study, and thus the direct effect of bIAP on LPS could not be assessed. Nonetheless, the specific activity of human placental alkaline phosphatase HPLAP and bIAP against an LPS insult has been undoubtedly demonstrated in vivo [16, 18]. Furthermore, it has been demonstrated that alkaline phosphatase dephosphorylates and thereby detoxifies not only endotoxins but also extracellular nucleotides [38]. Alkaline phosphatase converts these nucleotides into non-inflammatory nucleosines [39]. Both endotoxins and nucleotides are potent inflammatory triggers and are sensed as ‘stranger’or ‘danger’ signals to the innate immune system, and subsequent local and systemic inflammatory responses (SIRS) may result from the exposure to these pro-inflammatory signals [40, 41].

The physiological anti-inflammatory role of alkaline phosphatase as an important key factor in inflammatory insults has recently been confirmed in a model in zebra fish [42] as well as in a rat-enterocolitis model [43].

In conclusion, a single intravenous dose of bIAP reduced the production of the chymase mMCP-1 by mast cells and diminished the systemic pro-inflammatory cytokine response in the acute phase after AMI. Therefore, it is proposed that bIAP might represent a novel therapeutic drug in attenuating the pro-inflammatory response after AMI, thereby reducing the incidence of cardiogenic shock complications.
